# Chaperone-like chiral cages for catalyzing enantio-selective supramolecular polymerization†
†Dedicated to Professor Jean-Marie Lehn on the occasion of his 80th birthday.
‡
‡Electronic supplementary information (ESI) available. See DOI: 10.1039/c9sc02412c


**DOI:** 10.1039/c9sc02412c

**Published:** 2019-07-30

**Authors:** Yu Wang, Yibin Sun, Peichen Shi, Matthew M. Sartin, Xujing Lin, Pei Zhang, Hongxun Fang, Pixian Peng, Zhongqun Tian, Xiaoyu Cao

**Affiliations:** a State Key Laboratory of Physical Chemistry of Solid Surfaces , Collaborative Innovation Center of Chemistry for Energy Materials (iChEM) , Department of Chemistry , College of Chemistry and Chemical Engineering , Xiamen University , Xiamen 361005 , China; b Key Laboratory of Chemical Biology of Fujian Province , Xiamen University , Xiamen 361005 , China . Email: xcao@xmu.edu.cn ; Fax: +86 592 2186628 ; Tel: +86 592 2185862

## Abstract

Cage catalysis has emerged as an important approach for mimicking enzymatic reactions by increasing the reaction rate and/or product selectivity of various types of covalent reactions. Here, we extend the catalytic application of cage compounds to the field of non-covalent molecular assembly. Acid-stable chiral imine cages are found to catalyze the supramolecular polymerization of porphyrins with an accelerated assembling rate and increased product enantioselectivity. Because the imine cages have a stronger interaction with porphyrin monomers and a weaker interaction with porphyrin assemblies, they can fully automatically detach from the assembled products without being consumed during the catalytic process. We reveal the kinetics of the auto-detachment of cages and the chirality growth of the assemblies using spectroscopic characterization studies. We find that the passivation groups attached to the cages are important for maintaining the structural stability of the cages during catalyzed assembly, and that the steric geometries of the cages can profoundly affect the efficiency of chiral regulation. This strategy demonstrates a new type of catalytic application of cage compounds in the field of molecular assembly, and paves the way to controlling supramolecular polymerization through a catalytic pathway.

## Abstract 2

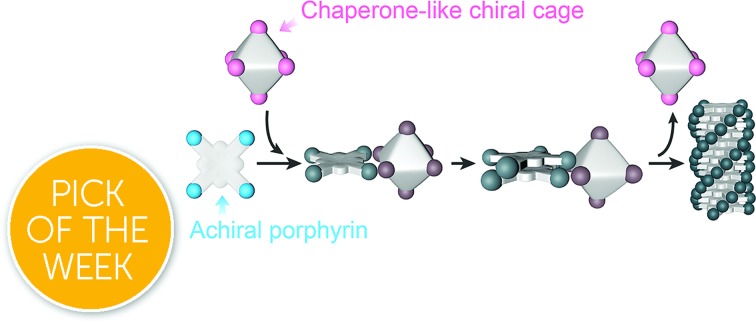
Chiral organic cages can assist enantio-selective supramolecular polymerization through a catalyzed assembly (catassembly) strategy, like chaperones assist the assembly of biomolecules.

## 1 Introduction

Cage compounds are discrete assemblies with sophisticated and well-defined local environments,^1^–^3^ making them an exceptional model for studying the fundamentals of molecular recognition^4^–^10^ and many related applications.^11^–^16^ For instance, cage catalysis has recently emerged as an important approach for mimicking enzymatic reactions by increasing the reaction rate and/or product selectivity of various types of reactions.^17^–^21^ As functional porous materials, cage compounds possess unique solution-processability for homogeneous catalysis,^1^ complementary with heterogeneous supramolecular catalysis through metal–organic frameworks^22^–^24^ or covalent organic frameworks.^25^–^27^ Extensive studies of cage catalysis have been conducted by using both metal–organic^28^–^31^ and pure organic cages.^32^–^35^ The catalytic strategies are versatile, and they include encapsulating the reactants or introducing functional groups either inside^36^–^38^ or outside^39^ the cage cavities. All these strategies are designed to realize the essential feature of cage catalysis: cages co-assemble with the reactants, and automatically release the resulting products after facilitating the reactions. To this end, the initial interaction between the cage and reactants needs to be strong enough to control the reaction pathway and hence to increase the reaction rate and/or selectivity. Once the product forms, its interaction with the cage weakens, due to increased steric hindrance, resulting in the automatic separation of the cage and product. Because such an intricate, dynamic series of interactions is required, rationally developing new systems or new models of cage catalysis remains a challenge.^40^–^43^

**Fig. 1:**
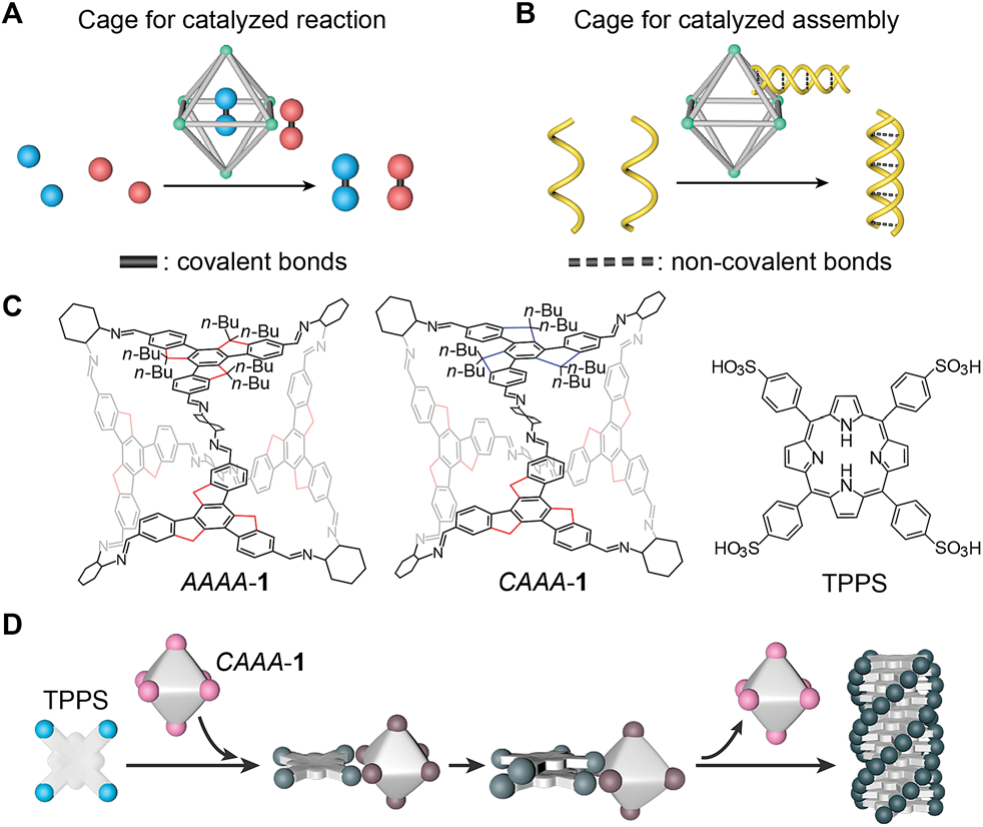
Schematic illustration and molecular structures of the cage-catalyzed molecular assembly. (A) Illustration of the cage-catalyzed strategy for covalent reactions. Coupling reactions can occur either inside or outside the cage cavity (still having interactions with the cage) as indicated by the red and blue balls, respectively. (B) Illustration of the cage-catalyzed strategy for non-covalent molecular assemblies. A cage assists the supramolecular recognition of two helical polymer chains, showing a novel catalytic mode for forming non-covalent bonds, in a way that resembles chaperone-assisted protein assembly. (C) Molecular structures of two isomers of cage **1**, *i.e.*, *CAAA*-**1** and *AAAA*-**1**, and the porphyrin TPPS. In the cage structures, butyl groups are only shown on one of four faces, for clarity. In complete structures, each sp^3^ carbon of the truxene backbones is connected to two *n*-butyl groups, *i.e.*, 24 butyl groups for a cage. Rotation patterns along the three sp^3^ carbons of the truxene backbones are either clockwise (*C*) or anticlockwise (*A*) when viewed from outside the octahedra, as indicated by the blue (*C*) and red (*A*) lines. (D) Schematic illustration of enantioselective supramolecular polymerization of TPPS catalyzed by *CAAA*-**1**. Note that the models show a reversible process on the cage vertices as indicated by the color change from pink to brown, and then back to pink in the final step. This illustrates how we can monitor the different stages of the catalyzed assembly process, using spectroscopy, and confirm the recovery of the cage after it catalyzes the formation of TPPS assemblies.

Here, we report a catalyzed assembly strategy to increase the assembly rate and enantio-selectivity of the supramolecular polymerization of porphyrins by using acid-stable chiral organic cages. A comparison between this strategy and the reported cage catalysis for covalent reactions is depicted in Fig. 1A and B. Both strategies share two important features, namely the increased efficiency and/or selectivity of a reaction or an assembly process, and the automatic release of products after the reaction. However, in the present study, cages facilitate the formation of non-covalent assemblies instead of covalent molecules, similar to the way chaperones assist the assembly of biomolecules.^44^,^45^ In addition to showing the chaperone-like behavior of cage compounds, we reveal the kinetics of the auto-detachment of cages and the chirality growth of the assemblies using spectroscopic characterization studies. Through comparison experiments with several cages and small molecules, we demonstrate that the passivation groups attached to the cages are important for maintaining the structural integrity of the cages during catalyzed assembly, and that their steric geometry can profoundly impact the chiral transfer between cages and assemblies.

## 2 Results and discussion

### 2.1 Molecular structures and schematic procedures of catalyzed assembly

Chiral imine cages were synthesized as previously reported,^46^*via* the condensation of four butylated truxene faces and six (*R*,*R*)-1,2-cyclohexanediamine (CHDA) chiral vertices (structures shown in Fig. 1C and the reaction schematic shown in Fig. S1A‡). Condensation at room temperature generates two isomers of face-rotating octahedra, which are named after the directionalities of the exterior faces as *AAAA*-**1** and *CAAA*-**1**. These cages possess excellent stability, allowing us to isolate them by chiral high-performance liquid chromatography (HPLC) and their individual structures have been confirmed by single-crystal X-ray diffraction (CCDC# 1406534 and 1406540).^46^ The isolated *T*-symmetric *AAAA*-**1** and *C*_3_-symmetric *CAAA*-**1** have very similar structures, except for the small differences in the arrangements of the butyl chains. These similarities enable close comparison between the complexes when they are used as catalysts. The structures of imine cages used for comparison experiments, including isomers of cage **2** with (*S*,*S*)-CHDA vertices, cage **3** with phenyl faces, cage **4** with ethylated truxene faces, and cage **5** with ethylenediamine (EDA) vertices, are shown in Fig. S1B–E.‡

Traditional supramolecular polymerization of tetrakis(4-sulfonatophenyl)-porphyrin (TPPS, structure shown in Fig. 1C) was selected as a representative non-covalent assembly system^47^–^55^ to be catalyzed by chiral cages. Supramolecular polymerization of TPPS has previously been regulated through a co-assembly method by adding oppositely charged chiral auxiliaries, such as amines^56^ and amino acids.^57^ Learning from the cage catalysts for covalent reactions, we consider that a stronger interaction with the reactant and weaker interaction with the product are the key to achieving the self-release of cages, and thus allows the recycling of cages after catalysis.

We envisioned that the chiral truxene imine cage **1** might be a potential candidate for catalyzing the enantioselective supramolecular polymerization of TPPS, because the chiral imine groups in cage **1** are less basic than the amine groups in (*R*,*R*)-CHDA, and hence they exhibit weaker interactions with the sulfonic groups in TPPS. Therefore, unlike the co-assembly of (*R*,*R*)-CHDA and TPPS,^56^ cage **1** and TPPS might not co-assemble in the final product, which is a necessary condition for achieving catalyzed assembly. In addition, the butyl chains on the outer surfaces of the cages provide stronger steric hindrance to the TPPS assemblies over the monomers,^46^,^58^ thus causing relatively stronger interactions with monomers and weaker interactions with assemblies (detailed in Fig. S2‡). This could lead to the automatic release of products from the cages after the enantioselective polymerization of TPPS (Fig. 1D).

### 2.2 Spectroscopic characterization of chiral supramolecular polymerization

To induce supramolecular polymerization, we add ethyl acetate, with or without imine cages, to the stock solution of TPPS in methanol to attain a solvent ratio of 9 : 1 (ethyl acetate : methanol, v/v). The self-assembly of TPPS occurs very slowly when the concentration of TPPS is 10 μM, showing no perceptible aggregation in two weeks. By contrast, when an equivalent amount of *CAAA*-**1** was introduced into this system, obvious precipitation was observed in 48 hours. Circular dichroism (CD) and UV-vis spectroscopy were used to show the catalytic behavior of *CAAA*-**1** for regulating the enantioselective supramolecular polymerization of TPPS.

**Fig. 2:**
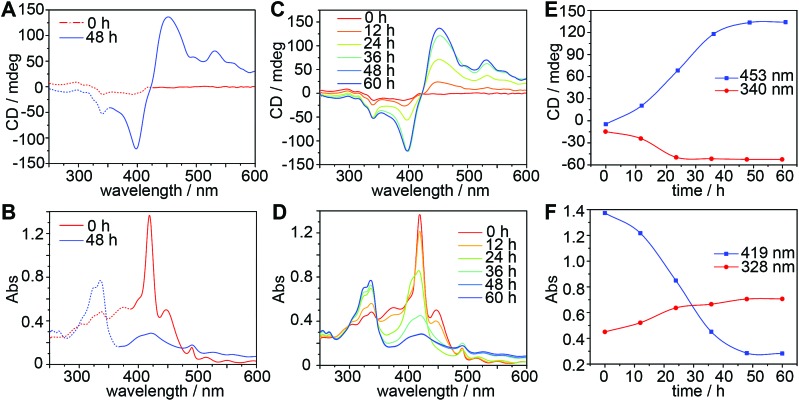
CD and UV-vis spectroscopic characterization of the catalyzed assembly of TPPS. (A and B) CD and UV-vis spectra of the equivalent mixture of TPPS and *CAAA*-**1** (10 μM) at 0 and 48 hours. Solid and dashed lines indicate the regions generated by TPPS assemblies and *CAAA*-**1**, respectively. (C and D) Real-time CD and UV-vis monitoring of the kinetics of the catalyzed assembly. (E and F) Time-dependent intensities of the representative peaks of CD and UV-vis spectra extracted from (C) and (D). Intensity increase of the CD peak at 340 nm and the UV-vis peak at 328 nm indicates the growth of free *CAAA*-**1**, the intensity increase of the CD peak at 453 nm indicates the growth of chiral TPPS H-assemblies, and the intensity decrease of the UV-vis peak at 419 nm indicates the consumption of TPPS monomers.

A CD spectrum recorded immediately after mixing TPPS and *CAAA*-**1** shows relatively weak and broad peaks at 340 nm and 400 nm (Fig. 2A), whereas the corresponding UV-vis spectrum shows the typical absorption band of the protonated TPPS monomer at 417 nm (Fig. 2B).^56^,^59^ The CD and UV-vis spectra of the TPPS and *CAAA*-**1** mixture at 0 hours are dramatically different from the sum of their individual spectra (Fig. S3‡), indicating that the interaction between TPPS and *CAAA*-**1** occurs immediately after mixing. After 48 hours, the formation of chiral TPPS H-assemblies was indicated by the strong exciton-coupled CD peaks at 400 nm and 453 nm and the corresponding UV-vis absorption peak at 419 nm (Fig. 2A and B). The chirality of the TPPS H-assemblies can be empirically assigned as right-handed based on the positive cotton effect in the CD spectrum.^47^,^56^,^59^ Meanwhile, the appearance of the typical peaks of *CAAA*-**1** in the CD spectrum (340 nm) and the absorption spectrum (328 nm) recorded at 48 hours, suggests that *CAAA*-**1** separated from the TPPS assemblies.

We further investigated the kinetics of the growth of TPPS assemblies and the release of the cage (Fig. 2C and D). As analyzed in Fig. 2E and F, the cage was almost completely released from TPPS after 24 hours. However, UV-vis spectra show continuously decreasing absorbance of the TPPS monomer at 417 nm, suggesting the further formation of TPPS assemblies from monomers and/or oligomers. During this process, the CD intensity of TPPS assemblies continues to grow until reaching its maximum at 48 hours. The chirality growth of the TPPS assemblies from 24 h to 48 h could be caused by the cage catalyst, and may also be facilitated by the previously formed chiral TPPS assemblies through self-propagation,^60^ as shown in Fig. S4.‡

To directly compare the catalyzed assembly with the self-assembly of TPPS, we increased the TPPS concentration from 10 μM to 50 μM. Under this condition, the self-assembly of TPPS took approximately 72 hours to show perceptible aggregation in solution (Fig. S5A‡). In contrast, after introducing 50 μM *CAAA*-**1**, obvious precipitation was observed in 30 min. UV-vis spectra show that TPPS molecules form J-assemblies in the self-assembly product and form H-assemblies when *CAAA*-**1** is added (Fig. S5B‡), indicating that *CAAA*-**1** can both accelerate the assembly of TPPS monomers and control the selectively of assembly products.

In addition, we have performed a concentration-dependent study on the cage catalysis as shown in Fig. S6.‡ In the systems with a consistent 10 μM concentration of TPPS, 10 μM *CAAA*-**1** results in the highest CD intensity of the TPPS H-assemblies. Increasing the concentration of *CAAA*-**1** decreases the CD intensity of TPPS assemblies, probably because a high concentration of the cage removes more protons from TPPS and thus causes stronger π–π interaction between TPPS molecules and relatively weakens the interaction between TPPS and *CAAA*-**1**, leading to a fast self-assembly process of TPPS with decreased chiral regulation from *CAAA*-**1**. For instance, aggregation in the 40 μM *CAAA*-**1** system is obvious after 1 hour, whereas it takes over 12 hours for the 10 μM *CAAA*-**1** system to show obvious aggregation. Decreasing the concentration of *CAAA*-**1** also leads to a decreased CD intensity of TPPS assemblies as less *CAAA*-**1** acts as the catalyst. We found the minimum concentration of the catalyst required to ensure the supramolecular polymerization of TPPS in our current experimental setup to be around 8 μM. When the concentration of *CAAA*-**1** goes lower than this limitation, *CAAA*-**1** starts to decompose, leading to poor reproducibility of the spectroscopic results. Note that this limitation is caused due to the hydrolytic decomposition of the cage, which is strongly related to the experimental conditions, especially the concentration of trace water in the system. We assume that the limitation could be further pushed towards lower concentrations if the trace amount of water can be reduced from the solvents and environments.

### 2.3 Recovery of the cage catalyst

**Fig. 3:**
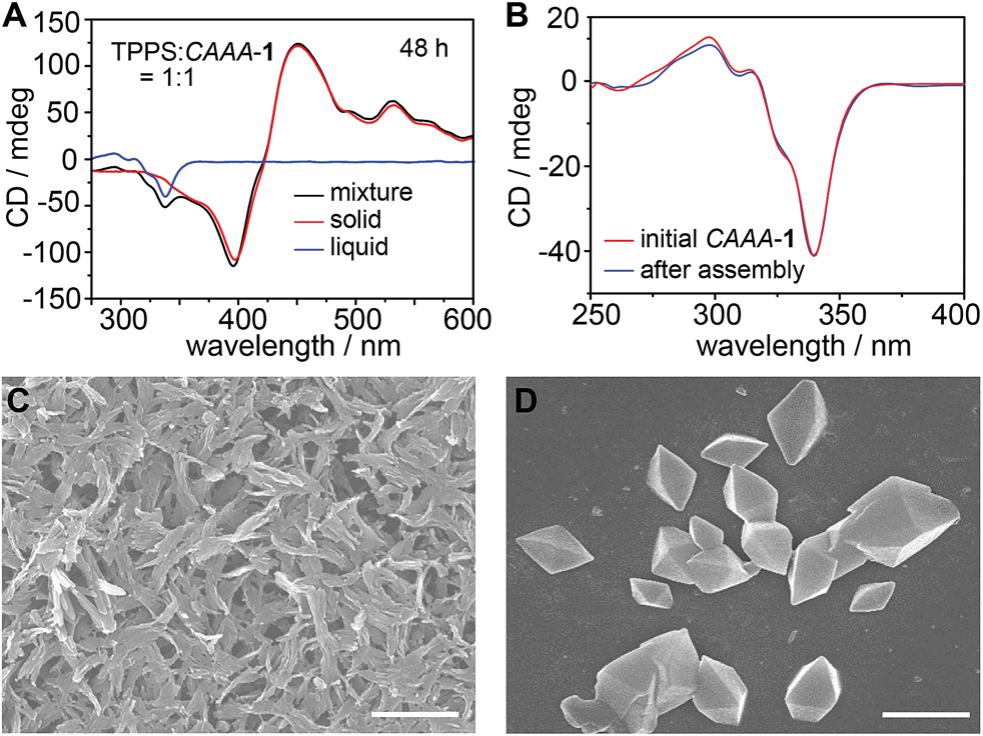
Auto-detachment of the cage catalyst revealed by CD and SEM characterization studies. (A) CD spectra of the precipitate (red), supernatant (blue), and thoroughly stirred mixture (black) of the assembly product of equivalent TPPS and *CAAA*-**1** at 48 hours, showing that TPPS and *CAAA*-**1** are completely separated into the precipitate and supernatant, respectively. (B) CD spectra of the initial *CAAA*-**1** (10 μM) and the supernatant after catalyzing the assembly of TPPS, showing the complete auto-detachment of the cage catalyst. (C and D) SEM images of TPPS assemblies (C) in the precipitate and *CAAA*-**1** crystals (D) obtained from the supernatant, showing completely different morphologies and their complete separation in the assembly product. Scale bars: 2 μm.

To further confirm the self-detachment of the cage, we characterized the isolated precipitate and the supernatant of the assembly product (TPPS : *CAAA*-**1** = 1 : 1, 10 μM) by CD, dynamic light scattering (DLS), and scanning electron microscopy (SEM). As shown in Fig. 3A, the CD spectra of the precipitate and the thoroughly stirred mixture overlap well in the region of TPPS assemblies, whereas the supernatant shows the typical spectrum of pure *CAAA*-**1**, without any signal from TPPS monomers or assemblies, suggesting that all TPPS assemblies have been precipitated from the solution. The CD spectrum of pure *CAAA*-**1** at 10 μM is almost identical to that of the supernatant (Fig. 3B), which confirms the full recovery of the cage catalyst after 48 hours. DLS shows that the formed TPPS assemblies are over 100 nm (Fig. S7‡). The SEM image of the precipitate further confirms that TPPS molecules have been assembled into nanorods and nanosheets with a corresponding diameter or thickness, respectively, of about 100 nm (Fig. 3C). The SEM image of the supernatant shows the octahedral shape of the cage single crystals (Fig. 3D), proving the purity of the cage in the supernatant.

Based on the above spectroscopic and SEM studies, we conclude that when the cage *CAAA*-**1** is introduced into the solution of TPPS, it interacts with TPPS immediately and thus increases the efficiency of supramolecular polymerization in terms of the assembly rate and the enantioselectivity of the product. After the formation of chiral TPPS assemblies, all cage molecules are completely restored to their initial states and can catalyze another round of supramolecular polymerization of TPPS. Therefore, we consider *CAAA*-**1** a chaperone-like self-detachable template for the enantioselective supramolecular polymerization of TPPS.

### 2.4 Acid stability of the truxene–CHDA imine cage

**Fig. 4:**
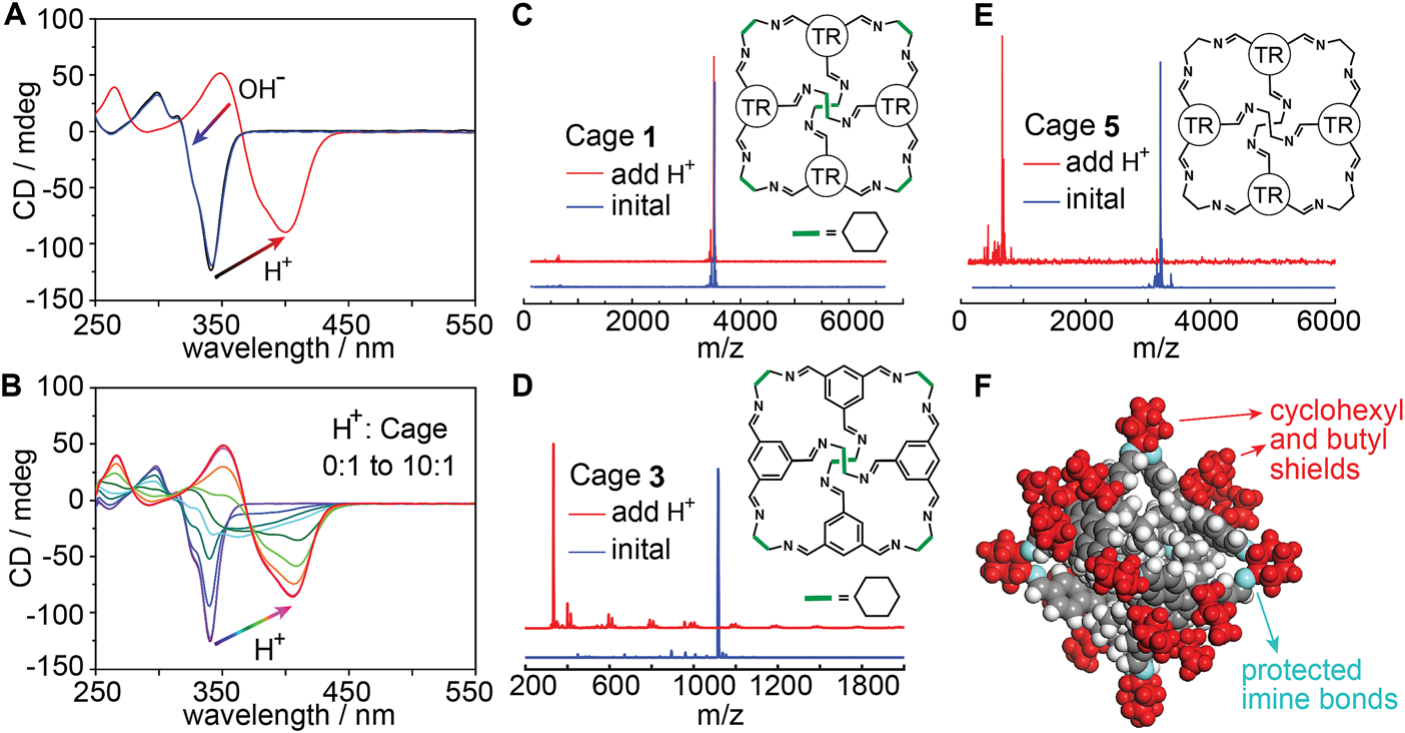
Extraordinary acid stability of the truxene imine cage. (A) CD spectra of *CAAA*-**1** (black) upon the addition of TFSA acid (red) and further addition of KOH base (blue). The amount of added TFSA and KOH is 10 equivalents to the initial *CAAA*-**1** (10 μM). (B) CD spectra of *CAAA*-**1** upon titration with TFSA. Note that the spectrum corresponding to a TFSA : *CAAA*-**1** ratio of 4 : 1 is very similar to the spectrum of the initial mixture of equivalent TPPS and *CAAA*-**1** (red dashed line in Fig. 2A). (C–E) Mass spectra of *CAAA*-**1** (10 μM) and two comparison imine cages **3** and **5** (10 μM) before (blue) and after (red) the addition of 10 equivalents of TFSA. TR groups in the inserted structures of **1** and **5** indicate the truxene faces with perpendicular butyl chains (detailed in Fig. 1C and S1‡). (F) Single-crystal structure of *CAAA*-**1** showing that the imine bonds are shielded by the outer cyclohexyl and butyl groups. The comparison of more cages and imine compounds in Fig. S9 and S10‡ shows that both the cyclohexyl and butyl shields and the cage shape are necessary for the extraordinary acid stability of cage **1**.

It is interesting that the absorption spectrum of *CAAA*-**1** exhibits weak and broad peaks after being mixed with TPPS and that it reverts to its original, intense peak at 340 nm after the separation from TPPS (Fig. 2A and C). We conjectured that this reversible spectroscopic change is related to the interaction between the acid groups in TPPS and the imine groups in the cage, *i.e.*, the protonation of the imine cage. To verify this conjecture, we studied the protonation of the cage under strong non-chromophore-containing trifluoromethanesulfonic acid (TFSA). As shown in Fig. 4A, upon adding 100 μM TFSA to 10 μM *CAAA*-**1** in ethyl acetate, the CD spectrum red-shifts to 400 nm immediately due to the protonation of the imine groups. Moreover, an additional 100 μM KOH can bring the CD spectrum fully back to its original shape. Similar reversible spectroscopic changes are also found in the fluorescence measurement (Fig. S8‡). Titration of TFSA with a *CAAA*-**1** solution gives a series of CD spectra showing the full range from the unprotonated to fully protonated states (Fig. 4B). The spectrum corresponding to a 1 : 4 ratio of cage : TFSA has a very similar shape to that of the spectrum of the 1 : 1 mixture of cage and TPPS (note that each TPPS monomer has four sulfonic groups), suggesting the partially protonated states of the cage during the initial stage of catalyzed assembly.

Mass spectroscopy further confirms that *CAAA*-**1** maintains the cage structure when the concentration ratio of TFSA to *CAAA*-**1** is 10 : 1 in ethyl acetate (Fig. 4C), showing extraordinary acid stability over other imine cages and imine-containing molecules under the same conditions. In controlled experiments, we have synthesized imine cages with similar [4 + 6] structures by changing the butyl-substituted truxene faces in cage **1** into unsubstituted phenyl faces (cage **3** in Fig. 4D and S1C‡) or ethyl-substituted truxene faces (cage **4** in Fig. S1D‡). Both cages **3** and **4** are decomposed into fragments under the same TFSA treatment (Fig. 4D, S9A and B‡). A similar decomposition occurs for cage **5** (Fig. 4E), which has ethyl vertices instead of the cyclohexyl vertices in cage **1**. Moreover, even though they consist of the same truxene and cyclohexyl components, the [1 + 1] condensation compound of butyl-substituted truxene and CHDA (structure shown in Fig. S10‡) still decomposes after the addition of TFSA, showing lower stability than the [4 + 6] cage counterpart. Imine bonds in all these structures have similar nucleophilicity, yet cage **1** exhibits the highest acid stability among them. This suggests that the cage shape and the passivating effects of cyclohexyl and butyl groups are both critical for protecting the imine bonds from decomposition (Fig. 4F), resembling the strategy of protecting soldiers with shields and a “testudo formation”.

### 2.5 Structural sensitivity to chiral transfer

**Fig. 5:**
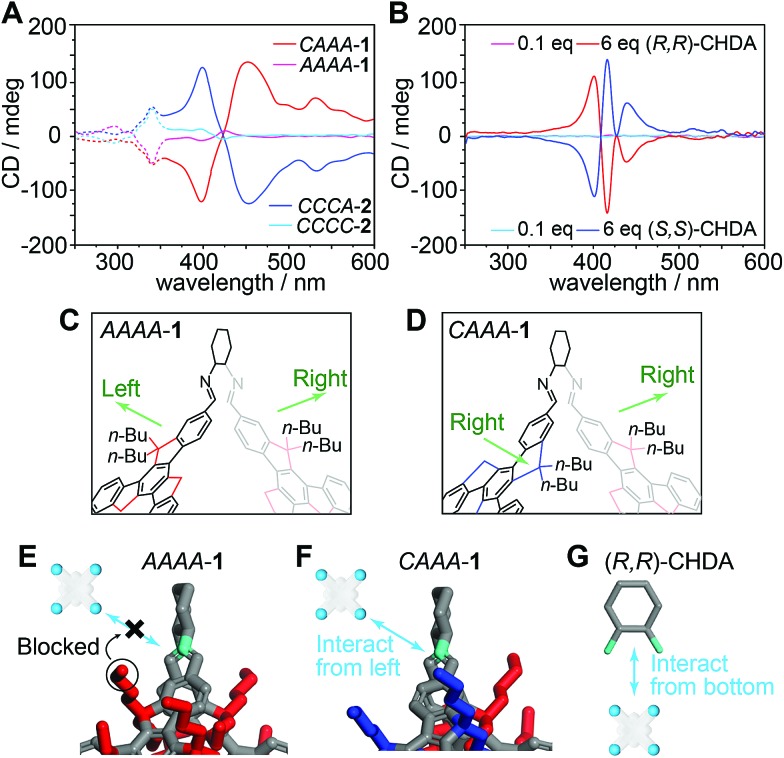
Structural sensitivity of chirality transfer from cages to TPPS assemblies. (A) CD spectra of TPPS assemblies catalyzed by truxene imine cages. Solid and dashed lines indicate the regions generated by TPPS assemblies and cages, respectively. *CAAA*-**1** and *AAAA*-**1** are formed from (*R*,*R*)-CHDA, whereas their mirror-image structures *CCCA*-**2** and *CCCC*-**2** are formed from (*S*,*S*)-CHDA. (B) CD spectra of TPPS co-assembled with (*R*,*R*)-CHDA and (*S*,*S*)-CHDA in different ratios. Note that the TPPS assemblies catalyzed by cage **1**, which has (*R*,*R*)-CHDA on the cage vertices, exhibit opposite chiroptical activity to the co-assemblies of TPPS and (*R*,*R*)-CHDA. The same chiral inversion is found between cage **2** and (*S*,*S*)-CHDA. (C and D) Structural details of *AAAA*-**1** and *CAAA*-**1** obtained by single-crystal X-ray analysis, emphasizing different arrangements of butyl chains on the vertices. For a vertex connecting two anticlockwise truxene faces (C), the two closest butyl groups point in opposite directions; for a vertex connecting a clockwise truxene face and an anticlockwise one (D), the two closest butyl groups point in the same direction. (E–G) Diagrams showing the different models of the interaction between TPPS and the imine in cage **1** or the amine groups in (*R*,*R*)-CHDA. It is difficult for *AAAA*-**1** to transfer its chirality to TPPS assemblies, due to strong steric hindrance in all directions. *CAAA*-**1** transfers its chirality to TPPS assemblies through the interaction in the least hindered direction, *i.e.*, the left. (*R*,*R*)-CHDA transfers its chirality to TPPS assemblies through the interaction in the least hindered direction, *i.e.*, the bottom.

We also perform the catalyzed assembly of TPPS by using the isomer *AAAA*-**1**, which has a very similar structure to *CAAA*-**1**, except for different rotational directions of one of the truxene faces and one of the related butyl groups. Therefore, we examine whether the steric geometry of truxene faces can influence the chiral transfer from the cage to TPPS assemblies. As shown in Fig. 5A and S11,‡ both *AAAA*-**1** and *CAAA*-**1** experience a similar protonated to deprotonated pathway during catalysis. However, *AAAA*-**1** exhibits a much lower efficiency for controlling the chirality of TPPS assemblies than does *CAAA*-**1**. After 48 hours, *AAAA*-**1** leads to chiral TPPS assemblies with a 10 mdeg peak in the CD spectrum, which is approximately 15 times lower than that generated by *CAAA*-**1**. In addition, we also synthesized imine cages with (*S*,*S*)-CHDA vertices, and isolated two cage products *CCCC*-**2** and *CCCA*-**2**, which are enantiomers of *AAAA*-**1** and *CAAA*-**1**, respectively. As expected, *CCCC*-**2** and *CCCA*-**2** generate mirror-like CD spectra compared with their enantiomers respectively (Fig. 5A).

Due to the dynamic nature of imine chemistry, it is possible that a tiny amount of cage **1** could hydrolyze and generate (*R*,*R*)-CHDA, and even we have not measured it through spectroscopic comparison (Fig. 3B). To make sure that the observed chiral catalysis is not an artifact phenomenon caused by partially hydrolyzed (*R*,*R*)-CHDA, we performed comparison experiments of the co-assembly of TPPS with (*R*,*R*)-CHDA in different concentrations. Since each *CAAA*-**1** contains six (*R*,*R*)-CHDA vertices, 6, 1, and 0.1 equivalents of (*R*,*R*)-CHDA correspond to the fully hydrolyzed, partially hydrolyzed, and barely hydrolyzed conditions of *CAAA*-**1**. The results show that both six equivalents or one equivalent of (*R*,*R*)-CHDA leads to the opposite chirality compared to one equivalent of *CAAA*-**1** (Fig. 5B and S12‡), whereas 0.1 equivalent of (*R*,*R*)-CHDA is insufficient to control the chirality of the TPPS assemblies. The chirality inversion between cages and their CHDA components rules out the assumption that the chiral regulation during catalyzed assembly is caused through chiral self-propagation by a trace amount of CHDA decomposed from cage vertices.

We scrutinize the structures of *AAAA*-**1**, *CAAA*-**1**, and their vertex component (*R*,*R*)-CHDA and propose a mechanism for their different catalytic efficiencies (Fig. 5C–F). Having a *T* symmetry, every CHDA vertex in *AAAA*-**1** connects to two anticlockwise truxene faces with their butyl chains pointing in both left and right directions (Fig. 5C). Therefore, there is a strong steric hindrance in both directions when TPPS or TPPS assemblies try to interact with the chiral vertices (Fig. 5E). By contrast, in *CAAA*-**1**, there are three CHDA vertices connected to an anticlockwise and a clockwise truxene face, with their butyl chains pointing in the same direction (Fig. 5D). Thus, *CAAA*-**1** can have a stronger interaction with TPPS and TPPS assemblies from the direction that is not blocked by the butyl chains (Fig. 5F). Compared with imine cage **1**, (*R*,*R*)-CHDA has more basic amine groups and much less steric hindrance. Therefore, it forms co-assemblies with TPPS instead of showing catalytic behavior.^56^ In addition, when interacting with TPPS, (*R*,*R*)-CHDA has a large steric hindrance in the direction of the cyclohexyl group (Fig. 5G), which is different from that in a cage. This may result in different chiral selectivities between imine cages and their CHDA counterparts.

## 3 Conclusion

To summarize, we have found acid-stable chiral imine cages for catalyzing the enantioselective supramolecular polymerization of TPPS. During the catalysis, chiral imine cages regulate the chirality of TPPS assemblies and serve as spectroscopic probes to allow monitoring of the assembly and self-detachment kinetics. This study provides a strategy to construct chiral supramolecular polymers from achiral building blocks through cage catalysis, which also entails efforts towards extending the applications of cage catalysts from covalent interactions to non-covalent molecular assembly. The concept to design a stronger interaction with monomers and a weaker interaction with assemblies is general for the development of this new type of catalysis in the field of molecular assembly. In addition, the “testudo formation” strategy in constructing acid-stable imine cages may inspire other designs for applications that require stable cages.^61^,^62^

## Conflicts of interest

There are no conflicts to declare.

## Supplementary Material

Supplementary information
