# Supplementary material for: Chaperone-like chiral cages for catalyzing enantio-selective supramolecular polymerization
Source: Chem Sci. 2019 Jul 30;10(35):8076–82. doi: 10.1039/c9sc02412c (PMC6910136; doi:10.1039/c9sc02412c)
Supplement: Supplementary file 1 [file SC-010-C9SC02412C-s001.pdf]

Electronic Supplementary Material (ESI) for Chemical Science.
This journal is © The Royal Society of Chemistry 2019

Supporting Information for

Chaperone-like chiral cages for catalyzing enantioselective supramolecular polymerization

Yu Wang,[1,†] Yibin Sun,[1,†] Peichen Shi,[1] Matthew M. Sartin,[1] Xujing Lin,[1] Pei Zhang,[1] Hongxun Fang,[1] Pixian Peng,[1] Zhongqun Tian,[1] and Xiaoyu Cao[1,2*]

[1]*State Key Laboratory of Physical Chemistry of Solid Surfaces, Collaborative Innovation Center of Chemistry for Energy Materials (iChEM), Department of Chemistry, College of Chemistry and Chemical Engineering, Xiamen University, Xiamen 361005, China.*
[2]*Key Laboratory of Chemical Biology of Fujian Province, Xiamen University, Xiamen 361005, China.*
[†]*Contributed equally to this work*
[*]*e-mail: xcao@xmu.edu.cn*

Contents

1 Supplementary Figures **2**

2 Supplementary Methods **15**

3 Supplementary Reference **17**

1 Supplementary Figures

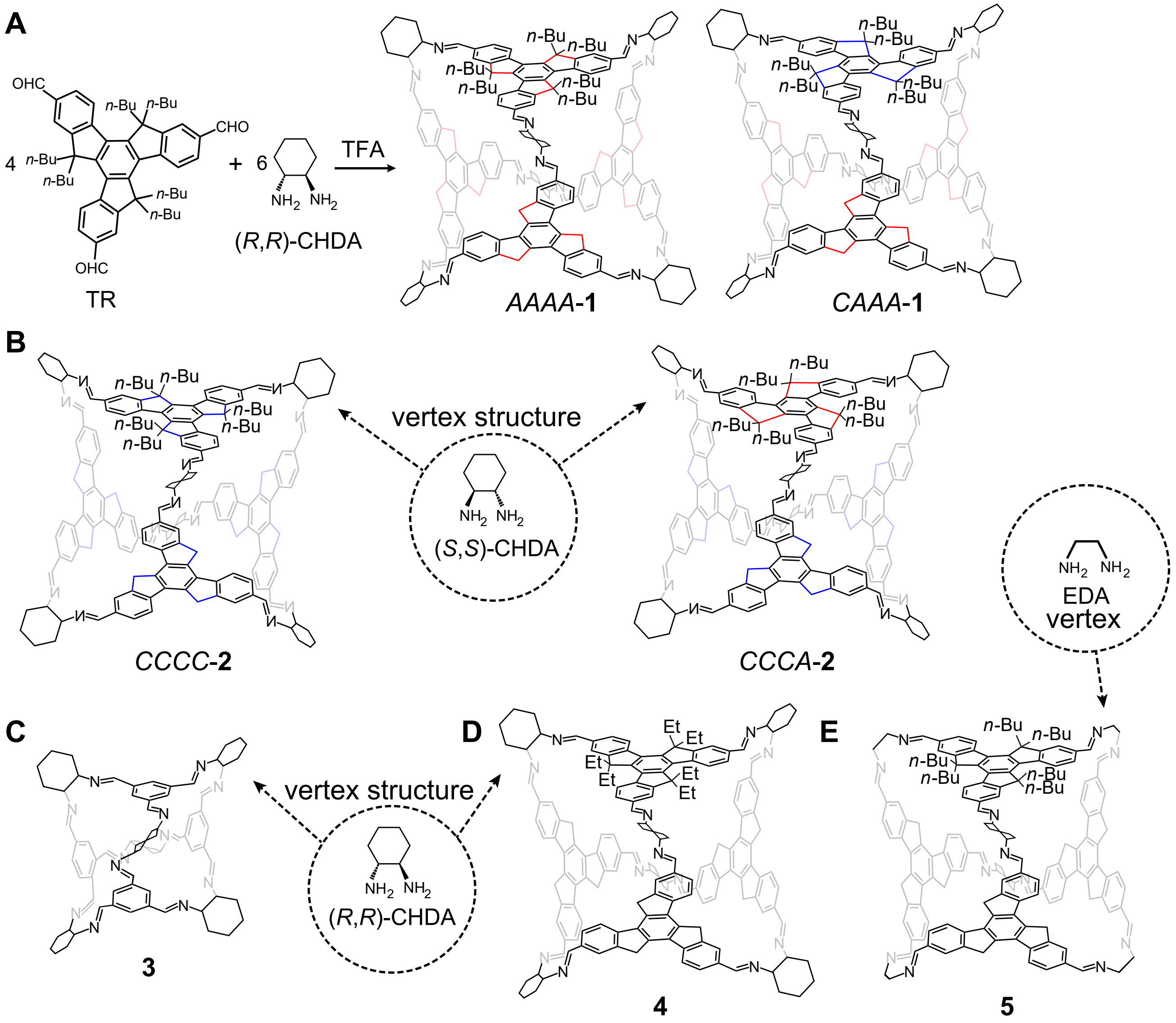

Figure S1: Schematic of synthetic route of cage 1 and molecular structures of other comparison cages. In the cage structures, butyl or ethyl groups are only shown in one of four faces for clarity. Rotation patterns along the three sp^3 carbons of the truxene backbones are considered to be either clockwise (*C*) or anticlockwise (*A*) when viewed from outside the octahedra, as indicated by the blue (*C*) and red (*A*) lines. (**A**) Cage **1** is synthesized through the condensation of four butylated truxene faces and six (*R*,*R*)-CHDA. Two isomers *AAAA*-**1** and *CAAA*-**1** are further isolated by chiral high-performance liquid chromatography (HPLC). (**B**) Cage **2** is synthesized through a similar procedure by using (*S*,*S*)-CHDA as the vertices. The resulting *CCCC*-**2** and *CCCA*-**1** are the mirror-image structures of *AAAA*-**1** and *CAAA*-**1**, respectively. (**C**) Structure of a smaller cage **3** synthesized through the condensation of four 1,3,5-triformylbenzene and six (*R*,*R*)-CHDA. (**D**) Structure of a ethylated truxene cage **4** synthesized from four ethylated truxene faces and six (*R*,*R*)-CHDA. (**E**) Structure of cage **5** with four butylated truxene faces and six EDA vertices. In all truxene cage structures, butyl groups are only shown in one of four faces for clarity. In complete structures, each sp^3 carbon of the truxene backbones connects to two butyl or ethyl groups. As shown in the comparison experiments (Figs. 4 and S10), only cages **1** and **2** (enantiomers) keep their structural integrity upon the addition of 10 times of TFSA, whereas cages **3**, **4**, and **5** decompose into monomers or fragments.

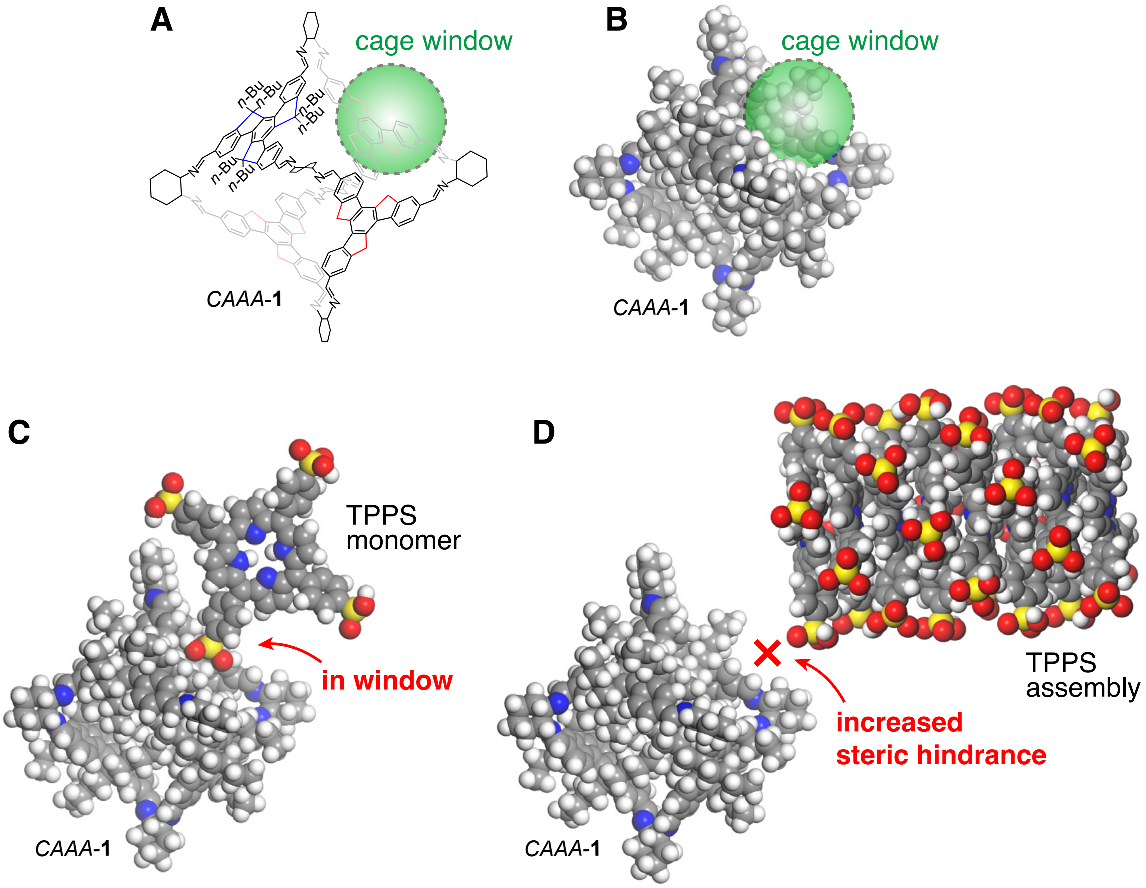

Figure S2: Comparison with the interaction between cage and TPPS monomer and the interaction between cage and TPPS assembly. (**A**) Chemical structure and (**B**) the space-filling model show the four uncapped windows of octahedral cage 1, providing cavities for guest molecules. (**C**) TPPS monomer can partially get into the cage windows, leading to a high affinity. (**D**) TPPS assembly cannot get into the cage windows due to the increased steric hindrance, leading to a lower affinity. Structures in B to D are built based on the single crystal structures of *CAAA*-**1** [1] and TPPS [2].

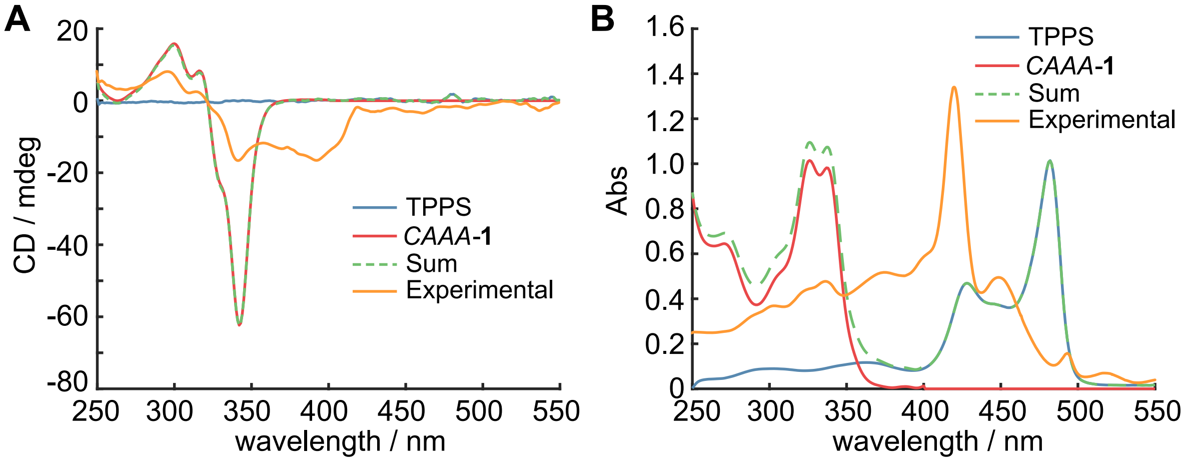

Figure S3: CD (A) and UV-vis (B) spectra of individual TPPS and *CAAA*-1 and their 1:1 mixture at 0 hour. Concentrations of all species are 10 μM. Dashed green lines represent the simply sum of individual TPPS (blue lines) and *CAAA*-**1** (red lines), whereas the orange lines represent the experimental measurements of the 1:1 mixture.

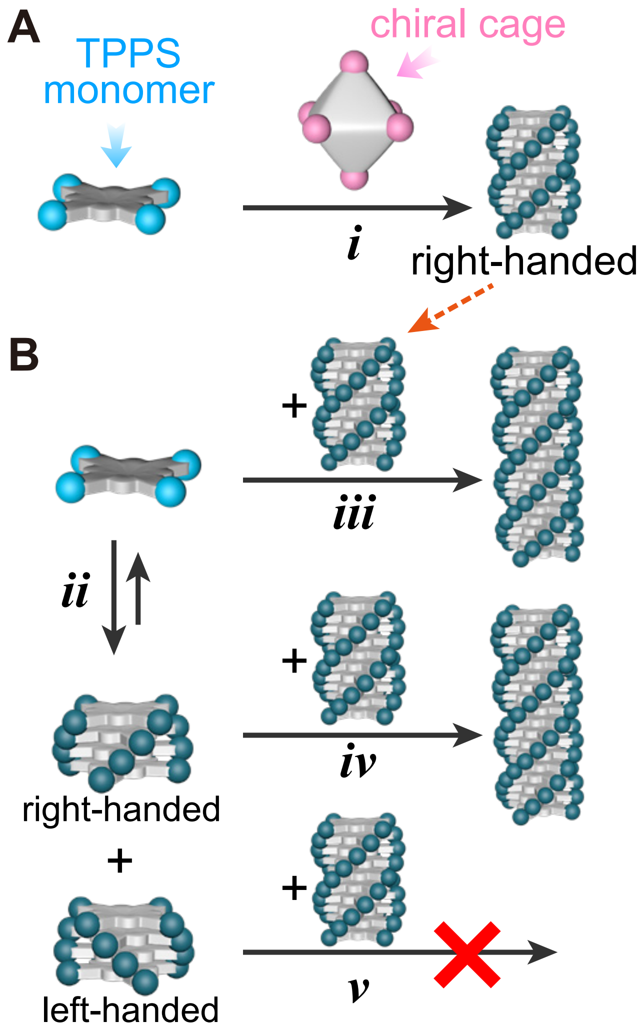

Figure S4: Schematic of the chirality growth during the supramolecular polymerization of TPPS. (**A**) In the initial stage, chiral cage *CAAA*-**1** helps the formation of right-handed TPPS assemblies in a catalytic pathway (indicated by arrow *i*) as detailed in Fig. 1D. (**B**) In parallel to the catalytic pathway (*i*), TPPS monomers can form right-handed and left-handed oligomers or assemblies through a spontaneous polymerization. For short oligomers, the assembling process is reversible, as shown by the double directional arrows in pathway **ii**. In the later stage of supramolecular polymerization (e.g., after 24 h), there are many right-handed assemblies existed in the system, which are formed in the early stage through the catalyzed pathway as indicated by the orange arrow between entries A and B. These right-handed assemblies can co-assemble with monomers (pathway *iii*) or right-handed oligomers (pathway *iv*) to achieve the self-propagation of right-handed assemblies. By contrast, the left-handed oligomers may not be able to connect with the right-handed assemblies, as shown by the arrow *v*. Overall, the pathways in entry B suggests that the self-propagation may also play a role in the chirality growth of supramolecular polymerization.

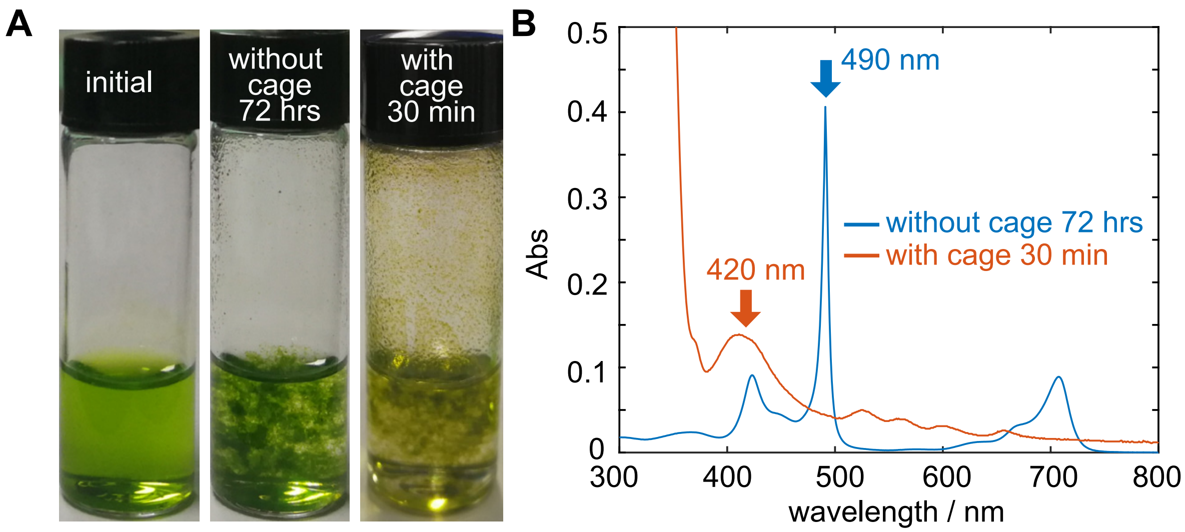

Figure S5: Comparison of the kinetics and products of the self-assembly and catalyzed assembly of TPPS. (**A**) Pictures of the initial solution of TPPS at 50 μM, self-assembled products at 72 hours, and the assembled product at 30 min catalyzed by 50 μM *CAAA*-**1**. (**B**) UV-vis spectra show a representative peak of J-aggregate of TPPS at 490 nm (blue arrow) for the self-assembled product and a representative peak of H-aggregate of TPPS at 420 nm (orange arrow) for the catalyzed product.

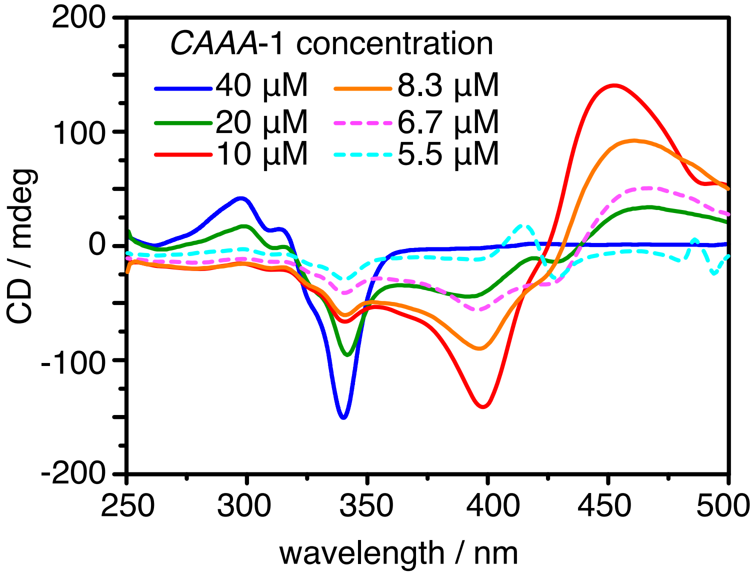

Figure S6: Concentration-dependent study on the cage catalysis. In all experiments, the concentration of TPPS was kept consistent of 10 μM, whereas the concentration of cage was altered accordingly. CD spectra of the mixtures were measured after 48 hours. As shown by Fig. 3B, comparing the CD spectra of initial *CAAA*-**1** and the *CAAA*-**1** after catalyzing the assembly allows us to evaluate whether *CAAA*-**1** is fully recovered after catalysis. We found the complete auto-detachment of intact *CAAA*-**1** when the concentration of *CAAA*-**1** is larger 8.3 μM (shown by the solid lines), however, *CAAA*-**1** starts to decompose at lower concentrations (shown by the dashed lines). The decomposition of *CAAA*-**1** generates (*R*,*R*)-CHDA, and the *CAAA*-**1** and (*R*,*R*)-CHDA co-exist in the partially decomposed system, e.g., with 6.7 μM *CAAA*-**1**. Therefore, the CD spectra of the partially decomposed system have a low reproducibility, because *CAAA*-**1** and (*R*,*R*)-CHDA compete in the system and complex kinetics are involved. In some case, the influence of (*R*,*R*)-CHDA could dominate and lead to the opposite chirality of the TPPS assemblies, e.g., cyan line with 5.5 μM *CAAA*-**1**. Note that, the minimum concentration of catalyst is caused due to the hydrolysis decomposition of cage, which is strongly related to the experimental condition, especially the concentration of trace water in the system. In order to yield a quantitatively reliable result on the hydrolysis of imine cages, one has to develop a method to further remove the trace amount of water from the chemicals and the HPLC grade solvents (used in this work) and also to quantitatively characterize the trace amount of water during catalyzed assembly.

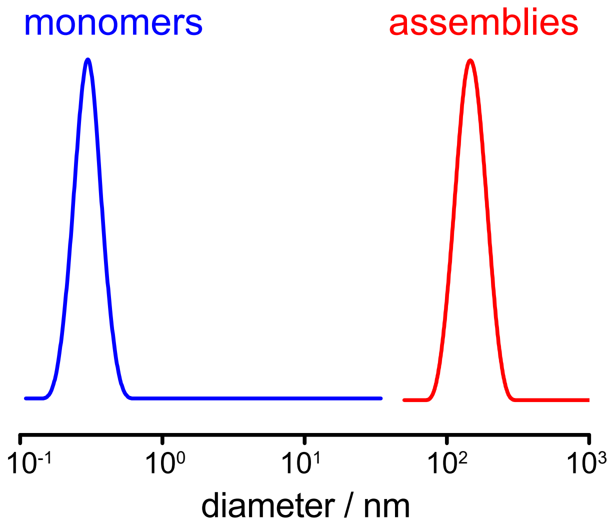

Figure S7: DLS spectra of TPPS monomers (blue) and TPPS assemblies (red) formed with the assistance of *CAAA*-1.

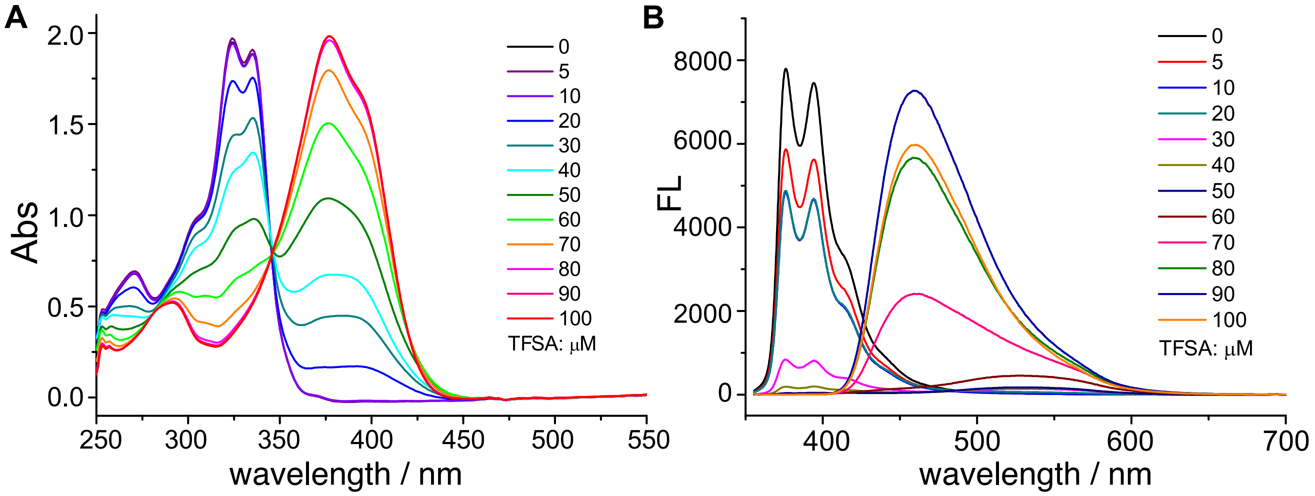

Figure S8: UV-vis (A) and fluorescence (B) spectra of *CAAA*-1 (10 μM in ethyl acetate) with the presence of different concentrations of TFSA.

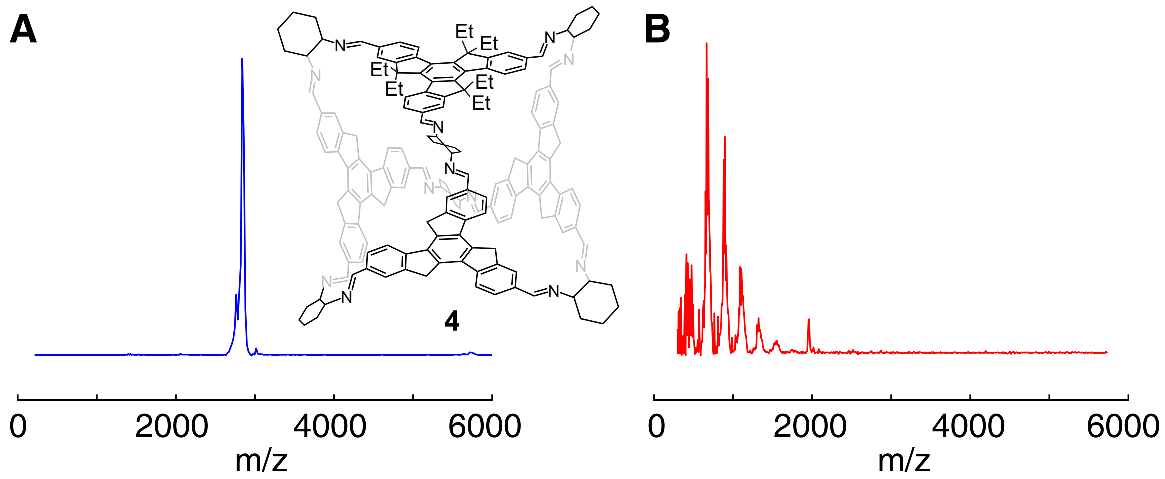

Figure S9: Decomposition of imine cages upon the addition of TFSA. MALDI-TOF mass spectra of cage **4** before (**A**) and after (**B**) introducing 100 μM TFSA into the cage solution (10 μM in ethyl acetate). The structural comparison with cage **1** is shown in Fig. S1.

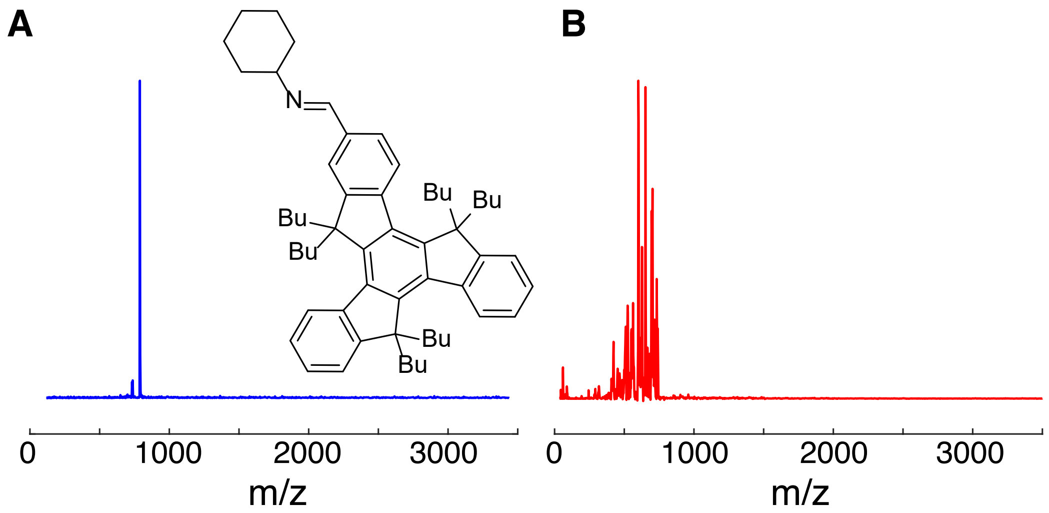

Figure S10: Decomposition of the [1+1] condensation compound of butyl-substituted truxene and cyclohexylamine. Structure of the [1+1] condensation compound is inserted and the synthesis procedure and structural characterization are shown in Fig. S13. Decomposition of the compound (10 μM in ethyl acetate) is shown by the MALDI-TOF mass spectra before (**A**) and after (**B**) the addition of 100 μM TFSA.

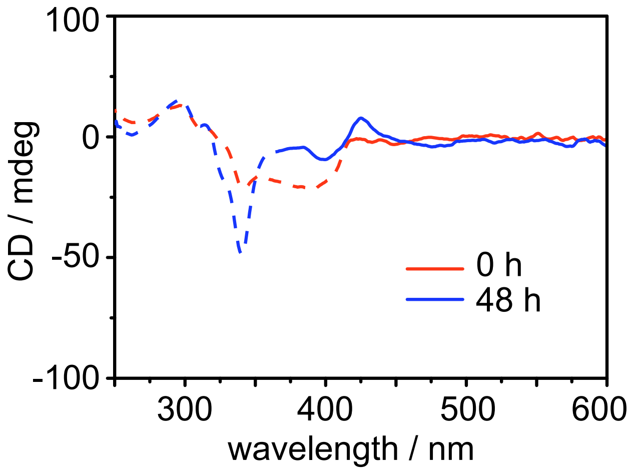

Figure S11: CD spectra of the 1:1 mixture of TPPS and *AAAA*-1 (10 μM) at 0 and 48 hours.

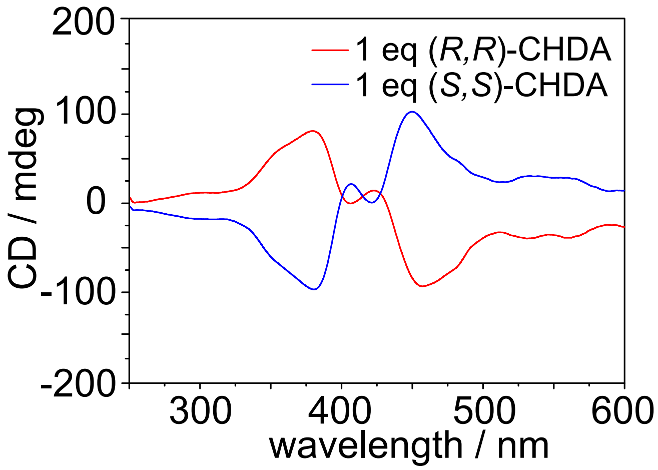

Figure S12: CD spectra of TPPS co-assembled with (*R*,*R*)-CHDA and (*S*,*S*)-CHDA in a 1:1 ratio.

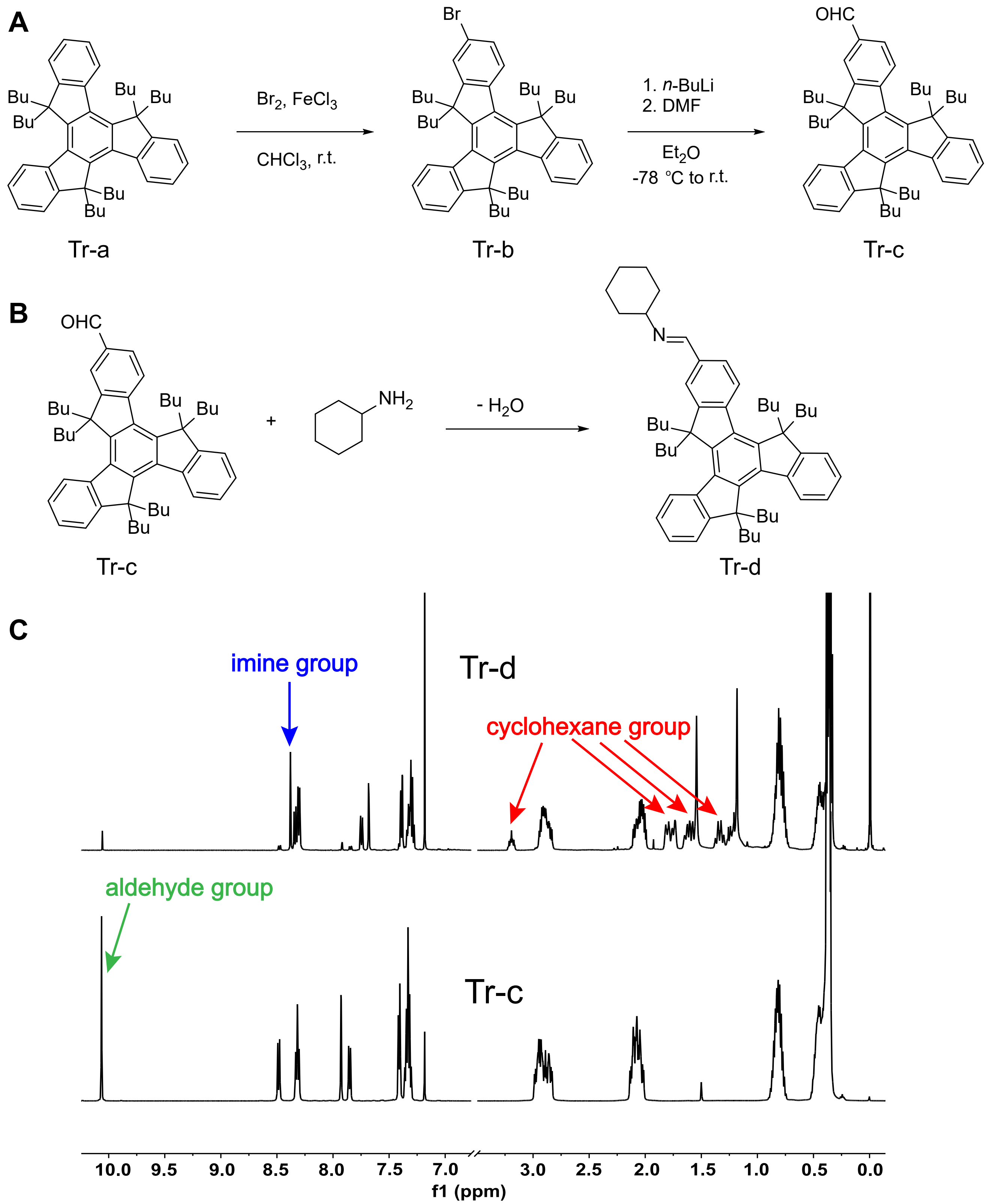

Figure S13: Synthesis and NMR characterization of the [1+1] condensation compound of butyl-substituted truxene and cyclohexylamine. (**A**) Synthesis procedure of mono-aldehydated truxene **Tr-c**. (**B**) Synthesis procedure of the [1+1] condensation compound **Tr-d**. (**C**) NMR spectra of **Tr-c** and **Tr-d**.

2 Supplementary Methods

Materials. Tetrakis(4-sulfonatophenyl)porphyrin (TPPS) was purchased from Dojindo Laboratories and used as received. Other reagents were all commercially available from Alfa, Sigma Aldrich and TCI, and used without further petrification. CD spectra were collected on JASCO J-810 circular dichroism spectrometer at 298 K. UV-vis spectra were collected on UV-2700 at 298 K. HPLC analyses were performed on a Shimadzu LC-16A instrument, using Daicel Chiralcel IE/IF Columns. Matrix-assisted laser desorption/ionization time of flight (MALDI-TOF) mass spectra (MALDI-TOF) were collected on a Bruker microflex LT-MS with 2,4,6-trihydrotyacetophenane (0.05 M in methanol) as matrix. SEM images were collected on a Hitachi S-4800 scanning electron microscope. ^{1}H and ^{13}C nuclear magnetic resonance (NMR) spectra were recorded on a Bruker AVIII-500 spectrometer (500 and 125 MHz, respectively) and are reported relative to residual solvent signals.

Syntheses and separations of cages. Synthesis of **1** and **2** follows the reported procedure[1] by reacting truxene aldehyde with (*R*,*R*)-CHDA or (*S*,*S*)-CHDA in a 1:1.5 ratio with catalytic amount of TFA. The small cage in Fig. S10 was synthesized by reacting benzene-1,3,5-tricarboxaldehyde with (*R*,*R*)-CHDA or (*S*,*S*)-CHDA in a 1:1.5 ratio with catalytic amount of TFA. HPLC separation of *CAAA*-**1** and *AAAA*-**1** was performed in the mobile phase of hexane:ethanol = 75:25 (v/v) with 0.1% diethylamine with Daicel Chiralcel IE Column. HPLC separation of *CCCA*-**2** and *CCCC*-**2** was performed in the mobile phase of methanol:methyl tert-butyl ether= 50:50 (v/v) with 0.1% diethylamine with Daicel Chiralcel IF Column.

Preparation of TPPS assemblies. In a typical procedure of cage catalyzed assembly, 1.8 mL ethyl acetate solution of chiral cage **1** (1.1×10^{-5} M) was injected into a TPPS methanol solution (0.2 mL, 1×10^{-4} M) in a 5 mL glass vial at 298 K. The solution was kept without disturbance until taken for CD or UV-vis measurements. Ratio of TPPS and *CAAA*-**1** was altered to 2:1, 1:2, and 1:4 by changing the concentration of *CAAA*-**1** accordingly, as shown in Fig. S6 . In the comparison experiments, cage **1** were replaced by other cages or by chiral CHDA with various concentrations.

Synthesis of mono-aldehydated truxene Tr-c. Procedure of the synthesis of is shown in Fig. S13A. **Tr-a** was obtained through a reported procedure[43]. To a stirred solution of **Tr-a** (5.0 g, 7.36 mmol) and $FeCl_3$ (10 mg) in $CHCl_3$ (100 mL), a solution of Br_2 (0.38 mL, 7.36 mmol) in $CHCl_3$ (20 mL) was added dropwise at room temperature. The reaction was kept stirring for 24 h and quenched with saturated sodium thiosulfate aqueous solution. The organic phase was washed with water and dried with anhydrous sodium sulfate. Solvents were evaporated under vacuum and crude product of **Tr-b** was obtained as yellow powder and used for next step. Yellow powder of **Tr-b** and anhydrous ethyl ether (100 mL) were added to a flask filled with nitrogen. The flask was stirred at -78°C and *n*-BuLi (12.27 mL, 29.44 mmol, 2.4 M in hexane) was added dropwise. After 0.5 h, the flask was warmed to room temperature and stirred for another 0.5 h. The reaction was then cooled to -78°C again and DMF (4.5 mL, 58.88 mmol) was added dropwise. The reaction was stirred overnight and quenched with aqueous HCl (2 M, 100 mL). Organic

phase was washed with water and dried over anhydrous sodium sulfate. Solvents were evaporated under vacuum and the solid was purified by column chromatography with petroleum ether: dichloromethane = 50:1 (v/v) to give **Tr-c** as white powder. **Tr-c**, ^{1}H NMR (500 MHz, $CDCl_3$, ppm, δ): 10.06 (s, 1H), 8.48 (d, J = 8.0 Hz, 1H), 8.34–8.29 (m, 2H), 7.93 (s, 1H), 7.85 (d, J = 8.0 Hz, 1 H), 7.41–7.40 (m, 2H), 7.36–7.30 (m, 4H), 3.00–3.82 (m, 6H), 2.14–2.00 (m, 6H), 0.90–0.73 (m, 12H), 0.52–0.32 (m, 30H). ^{13}C NMR (125 MHz, $CDCl_3$): 192.33, 154.55, 153.57, 153.44, 146.81, 146.21, 146.19, 139.87, 139.77, 139.04, 138.814, 137.09, 134.42, 129.30, 126.78, 126.75, 126.19, 124.83, 124.79, 124.69, 122.37, 122.30, 55.80, 55.72, 36.86, 36.56, 36.38, 26.55, 26.50, 22.83, 22.77, 13.80, 13.78. HRMS (m/z): $[M+H]^+$ calcd. for $C_{52}H_{66}O$, 707.51472; found, 707.52496.

Synthesis of the [1+1] condensation compound of butyl-substituted truxene and cyclohexylamine. Procedure of the synthesis of the [1+1] condensation compound **Tr-d** is shown in Fig. S13B. The NMR spectra of **Tr-c** and **Tr-d** are shown in Fig. S13C. Cyclohexylamine (7.01 mg, 0.0707 mmol) in 5 mL toluene and **Tr-c** (50 mg, 0.0707 mmol) in 5 mL toluene were added into a 40 mL vial and reacted at 90 °C for 3 days. After removing the solvents in vacuum, product was obtained as yellow solid and used without further purification. $[M+H]^+$ calcd. for $C_{58}H_{77}N$, 788.60896; found, 788.61196.

3 Supplementary Reference

[1] X. Wang, Y. Wang, H. Yang, H. Fang, R. Chen, Y. Sun, N. Zheng, K. Tan, X. Lu, Z. Tian and X. Cao, *Nat. Commun.*, 2016, **7**, 12469–12476.

[2] A. Fidalgo-Marijuan, E. Amayuelas, G. Barandika, B. Bazán, M. Urtiaga and M. Arriortua, *Molecules*, 2015, **20**, 6683–6699.
